# Supplementary material for: Improving Visual-Patient-Avatar Design Prior to Its Clinical Release: A Mixed Qualitative and Quantitative Study
Source: Diagnostics (Basel). 2022 Feb 21;12(2):555. doi: 10.3390/diagnostics12020555 (PMC8871093; doi:10.3390/diagnostics12020555)
Supplement: Supplementary file 1 [file diagnostics-12-00555-s001.zip › diagnostics-1542341-supplementary/diagnostics-1542341-supplementary.pdf]

Supplementary Material

Supplementary Material File S1: The four presented avatars, which were shown to the participants in the structured interviews of the first, qualitative study part. (**A**) demonstrates an awake patient with normal vital signs as would be found before administering general anaesthesia. In (**B**) the same patient state was shown but after general anaesthesia induction. This state differs from the first case by the lower bispectral index and the lower neuromuscular relaxation values. In (**C**) a hypotensive, desaturated, deeply sedated patient with a normal heart rate was shown. (**D**) indicates the condition of malignant hyperthermia in a patient under general anaesthesia (in particular high temperature and high end-tidal carbon dioxide (CO_2)).

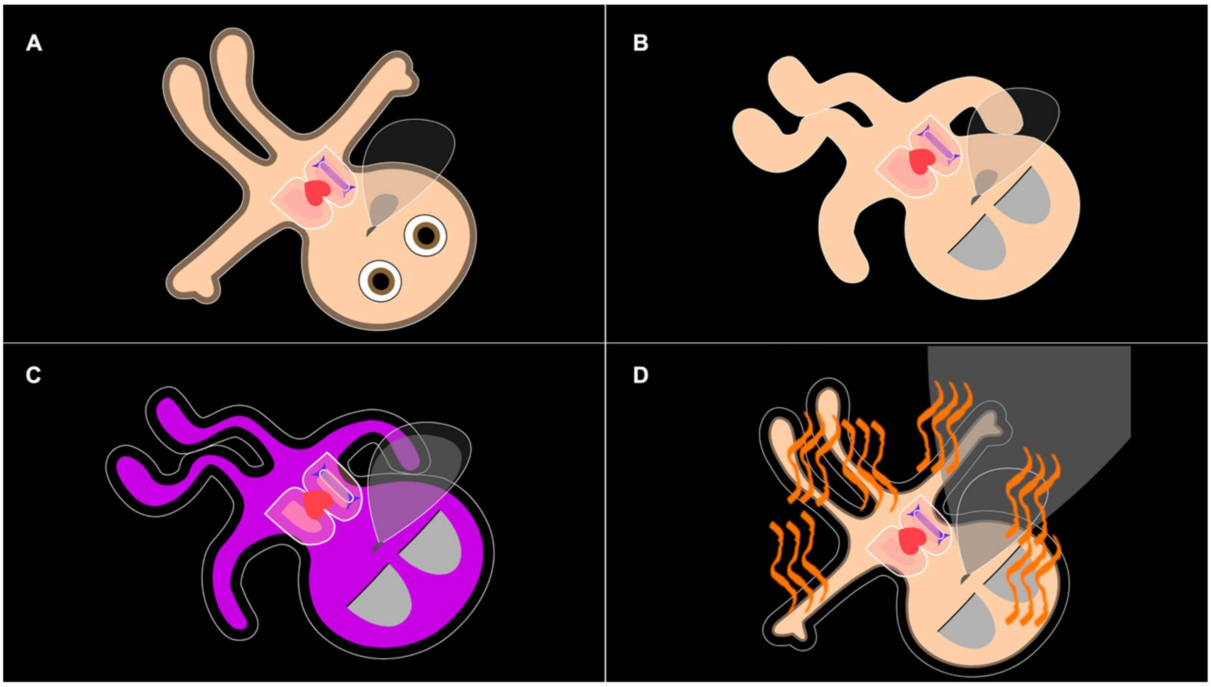

Supplementary Material File S2:

Exact Wording of the Standardised Instructions of the First, Qualitative Study Part.

The following introduction were provided to all participants of the first, qualitative study part: “You will shortly see a newly developed visualisation that attempts to represent the monitoring of vital signs as a simplified model. Please take a look at the following visualisations and try to say everything that goes through your mind”.

Supplementary Material File S3:

An Example Video of a Participant Interview of the First, Qualitative Study Part.

Link: https://1drv.ms/v/s!AjkumfX_cNxMi45b7ZW2rZ92LJboUw?e=rKdRIM

Supplementary Material File S4:

Transcription Rules for the Structured Interviews of the First, Qualitative Study Part.

Rule system adapted to our study conditions based on theories of Dresing* and colleagues.

It consists of three subsections: the content-semantic transcription rules, rules for extended content-semantic transcription, and notes on uniform spelling.

A. Content semantic transcription (= Basic rules for transcription)

1. A verbatim transcription is made, not phonetic or summarizing.

2. Word slurs are approximated to written German: For example, «So n Buch» is written as «so ein Buch», «hamma» is written as «haben wir».

3. The sentence form is retained even if it is syntactically incorrect.

4. Dialect expressions/ Swiss German is translated as literally as possible into High German, if no clear translation is possible, dialect is retained.

5. Colloquial particles such as "gell, ne" are transcribed literally.

6. Word breaks are marked with "/", e.g. “Heartbe/”. Semi-sentences that lack the completion are also marked with it, e.g. “He is only / “.

7. Stuttering is smoothed, but word duplications are also noted twice.

8. Punctuation is smoothed out in favour of readability, i.e., a period rather than a comma is used for brief lowering of the voice or ambiguous emphasis. Units of meaning should be retained.

9. Pauses are marked by (.), (..), (...), (....).

10. Especially emphasized words/expressions are marked by capital letters.

11. Each speaker contribution gets its own paragraph. There is a free, empty line between the different speakers.

12. Time stamps are inserted at the beginning of at least every paragraph, e.g. #01:29.

13. Emotional and/or nonverbal expressions (such as laughing, sighing, coughing, clearing the throat) are noted in brackets.

14. Incomprehensible words are marked with "(unv.)". Longer incomprehensible passages are marked with the cause if possible: "(unv. microphone rushes)". If one suspects a wording, the passage is put in brackets with a question mark, e.g. "(eye?)".

15. The interviewing person is indicated by "I:", the interviewee by a "B:".

16. If the change between the different visualizations is not announced by the interviewer or if the interviewee already makes a statement before the interviewer's announcement, an insertion with "First/Second/Third/Fourth Visualization" is inserted into the transcript at the appropriate place. If the visualization is announced, this insertion is placed before the announcement.

17. If background conversations take place by people other than the interviewer or interviewee that are overheard but not relevant to the content of the think aloud, this is not transcribed verbatim, but a note (background conversations) is made.

18. The file is named according to the media file name, e.g.: Transcript = Interview_034520.docx belonging to Audio file = Interview_ 034520.mp3.

B. Extended content semantic transcription: (For more in-depth representation by additionally capturing hesitations, word-finding inhibitions, etc.)

1. Pauses are marked by periods in brackets depending on their length. Here "(.)" stands for about one second, "(..)" for about two seconds, "(...)" for about three seconds and "(....)" for more than three seconds. For longer pauses a new paragraph with a new time marker can be started instead.

2. Reception signals and filler sounds ("hm, mhm, ja, aha, ähm" etc.) are transcribed.

3. After the particle "hm" a description of the emphasis is recorded in brackets, if this is clearly possible. The following are to be used: affirmative, negative, reflective, questioning, well-meaning, etc., e.g. "hm (affirmative)".

4. Several filler sounds in a row are typed without punctuation between them (e.g. "um um um so there would be ...").

C. Notes on uniform spelling (These points mainly affect the spelling and help to maintain a consistent style.)

1. The particles "hm" are always written "hm" regardless of the emphasis (not: hhmm, hmmm, hmh, etc.).

2. Sounds of hesitation are always written "um" (not: äm, ehm, öhm, etc.).

3. (Measurement) units are written out, e.g. meter, percent, etc..

4. Spoken signs are spelled out, e.g., at for @, and for &, etc..

5. Abbreviations are only typed if they are explicitly spoken that way ("etc." is only typed if "e te ce" is spoken).

6. Word contractions such as "runtergehen" instead of "heruntergehen" or "mal" instead of "einmal" are written exactly as they are spoken.

7. English or other foreign language expressions are treated according to German spelling rules in upper and lower case.

8. Personal pronouns of the second person (du, ihr) are written in lower case, personal pronouns of the polite form (Sie and Ihnen) are written in upper case.

9. Figures of speech/idioms are rendered literally, e.g. «übers Ohr hauen» (instead of: «über das Ohr hauen»).

10. Single letters are always capitalized.

11. Numbers are presented as follows:

a. Numbers from zero to twelve in continuous text as words, larger ones in digits.

b. Other numbers with short names are also written out, especially round ones:

twenty, one hundred, three thousand, etc..

c. Decimal numbers, mathematical equations, numerical values of units are always to

be written in digits. Thus: "4+5=9", "3.5", etc.

Reference:

*Dresing T, Pehl T, Schmieder C. Manual (on) Transcription. Transcription Conventions, Software Guides and Practical Hints for Qualitative Researchers (3rd English Edition) Marburg, Germany, 2015. Available from: http://www.audiotranskription.de/english/transcription-practicalguide.htm (accessed August 21, 2021)

Supplementary Material File S5:

Exact Wording of the Introduction to the Survey of the Third, Quantitative Study Part.

All participants of the third, quantitative study part were provided with the following introduction: "Today you are taking part in a study on the Visual Patient (VP). Before we begin, here is a brief introduction to what the Visual Patient is all about. The VP is a completely new form of visualisation of patient monitoring data. It represents the measured vital parameters in a simplified way, not as usual in numerical form, but pictorially in the form of a figurative patient. The VP was invented by David Tscholl, MD, and developed together with his research group and industry partner Philips. The declared goal is to establish the VP in the clinic in order to show changes in vital parameters more concisely and thus to promote situational awareness. This should ultimately lead to more rapid adequate and thus better treatment of patients. After the last large VP simulation study in summer 2020, the design was completely revised. This new design will now be tested in the current study."

Supplementary Material File S6:

An Example Video of the Third, Quantitative Study Part.

Link: https://1drv.ms/v/s!AjkumfX_cNxMi45Y3Lk2iMpb4j-L2g?e=I5E2Wa

Supplementary Material File S7:

Table 1. Vital sign identifications in the transcripts of the first, qualitative study part. Results summarised for all four visualisations. Statement counts with percentage of the total of given statements per vital sign. N4 = N1+N2+N3. The total of the given statements is 756.

Vital Sign	Correct Interpretation; N1 (%)	Incorrect Interpretation; N2 (%)	Unspecific Mentioning; N3 (%)	Total Given Statements per Vital Sign; N4 (% of All Given 756 Statements)
Arterial Blood Pressure	27 (44%)	8 (13%)	26 (42%)	61 (8%)
Pulse / Heart Rate	42 (26%)	49 (31%)	66 (42%)	157 (21%)
Central Venous Pressure	5 (10%)	41 (80%)	5 (10%)	51 (7%)
Oxygen Saturation	34 (37%)	6 (7%)	52 (57%)	92 (12%)
Temperature	38 (76%)	9 (18%)	3 (6%)	50 (7%)
Bispectral Index	44 (37%)	3 (3%)	72 (60%)	119 (18%)

Neuromuscular Relaxation	25 (42%)	17 (28%)	18 (30%)	60 (8%)
End-tidal CO_2	8 (8%)	32 (33%)	57 (59%)	97 (13%)
Tidal Volume	8 (19%)	4 (9%)	31 (72%)	43 (6%)
Respiratory Rate	9 (35%)	12 (46%)	5 (19%)	26 (3%)

Supplementary Material File S8:

Complete dataset for the vital sign identification analysis.
